# Synergistic Inhibition of Mycotoxigenic Fungi and Mycotoxin Production by Combination of Pomegranate Peel Extract and Azole Fungicide

**DOI:** 10.3389/fmicb.2019.01919

**Published:** 2019-08-20

**Authors:** Sudharsan Sadhasivam, Orr H. Shapiro, Carmit Ziv, Omer Barda, Varda Zakin, Edward Sionov

**Affiliations:** ^1^Department of Food Quality and Safety, Institute for Postharvest and Food Sciences, Agricultural Research Organization, The Volcani Center, Rishon LeZion, Israel; ^2^Department of Postharvest Science, Institute for Postharvest and Food Sciences, Agricultural Research Organization, The Volcani Center, Rishon LeZion, Israel

**Keywords:** pomegranate peel extract, prochloraz, mycotoxigenic fungi, combination treatment, aflatoxin B1, synergistic interaction

## Abstract

Fungal plant pathogens cause considerable losses in yield and quality of field crops worldwide. In addition, under specific environmental conditions, many fungi, including such as some *Fusarium* and *Aspergillus* spp., are further able to produce mycotoxins while colonizing their host, which accumulate in human and animal tissues, posing a serious threat to consumer health. Extensive use of azole fungicides in crop protection stimulated the emergence of acquired azole resistance in some plant and human fungal pathogens. Combination treatments, which become popular in clinical practice, offer an alternative strategy for managing potentially resistant toxigenic fungi and reducing the required dosage of specific drugs. In the current study we tested the effect of pomegranate peel extract (PPE) on the growth and toxin production of the mycotoxigenic fungi *Aspergillus flavus* and *Fusarium proliferatum*, both alone and in combination with the azole fungicide prochloraz (PRZ). Using time-lapse microscopy and quantitative image analysis we demonstrate significant delay of conidial germination and hyphal elongation rate in both fungi following PPE treatment in combination with PRZ. Moreover, PPE treatment reduced aflatoxin production by *A. flavus* up to 97%, while a combined treatment with sub-inhibitory doses of PPE and PRZ resulted in complete inhibition of toxin production over a 72 h treatment. These findings were supported by qRT-PCR analysis, showing down-regulation of key genes involved in the aflatoxin biosynthetic pathway under combined PPE/PRZ treatment al low concentrations. Our results provide first evidence for synergistic effects between the commercial drug PRZ and natural compound PPE. Future application of these findings may allow to reduce the required dosage of PRZ, and possibly additional azole drugs, to inhibit mycotoxigenic fungi, ultimately reducing potential concerns over exposure to high doses of these potentially harmful fungicides.

## Introduction

Many fungal plant pathogens that belong to the genera *Aspergillus* and *Fusarium* produce important mycotoxins of concern in relation to animal and human health (Tsitsigiannis et al., 2012). These fungi represent serious phytopathological and mycotoxicological risks at pre- and post-harvest stages, as well as in processed food products (Castoria et al., 2008). Mycotoxins, which are secondary metabolites produced by these fungi, have a significant economic impact worldwide as they pose a significant threat to food and feed safety, as well as in medical settings. Indeed, among natural food and feed contaminants, mycotoxins represent one of the major concerns regarding chronic toxicity, and pose critical challenges in food toxicology (Dellafiora and Dall’Asta, 2017). Although much progress has been made toward developing different agents to control mycotoxigenic pathogens at pre- or post-harvest stages, the number of efficacious antifungal drugs that can be used in food-production setting remains limited. Of these, azole-based fungicides are the most used antifungals in agriculture, due to their high efficiency and broad spectrum activity (Price et al., 2015). Thousands of tons of azoles are sold annually to control fungal infections in crops. According to the instructions of manufacturers, about 10 mg of azoles should be applied per 1 m^2^ of the field (Hof, 2001). Excessive and long-term use of azole fungicides in agriculture has led to the emergence of acquired azole resistance in some plant pathogenic fungi (Serfling et al., 2007). Moreover, several recent studies demonstrated that exposure of *Aspergillus* species, especially.

*Aspergillus fumigatus*, to azole compounds in the environment can induce cross-resistance to medical azole drugs (Chowdhary et al., 2013; Ren et al., 2016; Verweij et al., 2016). The development of drug resistance in many fungal pathogens, as well as growing public concerns over the health and environmental impacts of fungicides, has led to a significant interest in the development of alternative, environmentally friendly methods of disease control. Plant extracts are generally considered environmentally safer (i.e., biodegradable with low toxicity to the environment) and thus preferable alternatives to synthetic compounds. Plants produce a wide diversity of secondary metabolites which serve them as defense compounds for their own protection against other plants, pests and microbes. Several plant extracts were reported to exhibit a direct antifungal activity in treated plant hosts (Tripathi and Dubey, 2004; Palou et al., 2016). These secondary metabolites exhibit a wide range of biological and pharmacological properties, leading to the use of several products isolated from plants in the treatment of microbial infections in a number of host-pathogen combinations (Wink, 2015). Combining different antifungal compounds with different modes of action could reduce the required dose of each drug while minimizing the potential for the development of drug-resistance, still allowing for effective combating of fungal infections. While a number of recent studies explored the interactions between natural products and antifungal drugs (Shin, 2003; Shin and Kang, 2003; Shin and Lim, 2004; Karioti et al., 2011), the use of such combined antifungal treatments in agricultural setting remains limited, particularly when compared to clinical applications. Pomegranate by-products, such as peel and seeds, are considered a rich source of bioactive compounds such as flavonoids, phenolic acids, and tannins which have free radical scavenging activity and antioxidant capacity (Panichayupakaranant et al., 2010; Sorrenti et al., 2019). Several studies have been reported on the effectiveness of pomegranate peel extracts (PPEs) against human and plant fungal pathogens (Dikmen et al., 2011; Glazer et al., 2012; Foss et al., 2014; Pangallo et al., 2017; Rosas-Burgos et al., 2017). However, harmful fungi often have greater tolerance to such natural compounds when compared to commercially available antifungals. Several additional factors, such as low curative effect, reduced and inconsistent efficacy, and limited range of antifungal activities, represent major barriers to the commercial acceptance of plant extracts and other natural products for controlling agriculturally relevant fungal pathogens (Campbell et al., 2012; Bautista-Baños et al., 2013). The development of treatments combining natural compounds with commercial antifungal drugs is a promising approach toward harnessing the power of naturally occurring compounds. Considering the limited number of antifungal agents available, and that most of them have similar modes of activity (Loeffler and Stevens, 2003), their combination with natural antifungals, possibly with different modes of activity, has the potential for synergistic interaction. In the present study we evaluated an antifungal activity of PPE, and its potential for synergistic combination with an agricultural azole drug prochloraz (PRZ). We show that this combination is highly effective at inhibiting growth of the most prevalent mycotoxigenic fungal pathogens and mycotoxin production, suggesting a potential for such an approach in improving food and feed safety.

## Materials and Methods

### Preparation of Pomegranate Peel Extract

Pomegranate fruits (*Punica granatum* L.) from the “Wonderful” variety were purchased from local markets. Fruits were washed, and the arils were manually removed. The fruit peels were cut, frozen at −80°C, lyophilized and milled into a fine powder using an electric blender. The dried powder (100 g) was extracted with 500 ml of 80% methanol for 72 h at room temperature in the dark. The extract PPE was filtered through Whatman No. 1 filter paper, concentrated using a rotary evaporator (Buchi R-100, Switzerland) at 45°C, freeze-dried and kept at −20°C until use. PPE was dissolved in dimethyl sulfoxide (DMSO) at 100 mg/ml and kept at −20°C.

### Fungal Strains, Media, Growth Conditions, and Chemicals

*Aspergillus flavus* (NRRL3518) and *F. proliferatum* (NRRL31866) were used throughout the study. In some susceptibility tests also *A. parasiticus* (NRRL6111), *A. fumigatus* (NRRL62427), and *F. verticillioides* (NRRL25457) were used. The isolates were obtained from USDA Agricultural Research Service Culture Collection (Northern Regional Research Laboratory, Peoria, IL, United States). Strains were refreshed from −80°C by sub culturing on solid potato dextrose agar (PDA; 0.4% potato starch, 2% dextrose, and 2% agar) or broth (PDB) and maintained on PDA plates at 28°C before each experiment. Conidia were collected in sterile saline and the conidial suspension was adjusted to the required concentration by counting in a hemocytometer. The inoculum of the test strains was verified by plating on PDA plates for determination of colony forming units (CFU) counts. PRZ (Sigma) was prepared in DMSO at 25 mg/ml; stock solution was kept at −20°C. RPMI 1640 medium (Sigma) buffered with 0.165M MOPS (morpholinepropanesulfonic acid; pH 7) was used for antifungal microdilution susceptibility testing.

### Antifungal Susceptibility Testing

The *in vitro* activities of the antifungal compounds against mycotoxigenic fungi were determined using the standardized CLSI M38-A2 broth microdilution method (CLSI, 2008), with slight modification. Briefly, antifungal agents were dispensed in 96-well microtiter plates with two-fold serial dilutions of compound. The final compound concentration was prepared from stock solution in RPMI 1640 medium. The concentration of PPE and PRZ in the wells ranged from 9.76 to 5000 μg/ml and 0.0078 to 4 μg/ml, respectively. The stock conidial suspension (10^6^ spores/ml) was diluted to a final inoculum concentration of 0.4 × 10^4^ to 5 × 10^4^ spores/ml and dispensed into the microdilution wells. Minimal inhibitory concentrations (MIC) of the compounds were determined after 48 h incubation at 28°C. The MIC value was considered as the lowest compound concentration with no visible growth. Interactions between PPE and azole drug were assessed by checkerboard assays to determine the fractional inhibitory concentrations (FIC) of the combination of PPE and PRZ (Meletiadis et al., 2003). The first compound of the combination PPE was serially diluted along the abscissa (horizontal, *x*-axis), while the second drug PRZ was diluted along the ordinate (vertical, *y*-axis). Each microtiter well was inoculated with 100 μl of a fungal inoculum (0.4 × 10^4^ to 5 × 10^4^ conidia/ml), and the plates were incubated at 28°C for 48 h. The resulting checkerboard contains each combination of two compounds, with wells that contain the highest concentration of each compound at opposite corners. The FIC of each compound was calculated by using both MIC endpoints as described previously (CLSI), namely, the ratio of the concentration of the drug in combination that achieves the MIC endpoint to the MIC of the drug alone by using that endpoint. The FIC index (FICI) value was calculated by adding the FIC of PPE to the FIC of PRZ. Drug interactions were classified as follows: FICI ≤ 0.5, synergistic; 0.5 < FICI ≤ 1, additive; 1 < FICI ≤ 4, indifferent; FICI > 4, antagonistic.

### Live Imaging Microscopy

*Aspergillus flavus* and *F. proliferatum* were treated with PPE and PRZ alone, and in combination (checkerboard method), and examined under live imaging microscope. PPE and PRZ were serially diluted in 24-well microtiter plate at the concentration ranges of 156.25 to 2500 μg/ml and 0.0625 to 0.25 μg/ml, respectively. Each well containing each drug alone and combination of two compounds was inoculated with 200 μl of a fungal inoculum of 0.4 × 10^5^ to 5 × 10^5^ conidia/ml. The plate was placed on a motorized stage and conidial germination and hyphal growth were monitored for 24 h at 28°C under a live imaging microscope. Different parameters, such as time to germination, inhibition of conidial germination, mean hyphal elongation rate and maximum hyphal length, were determined for assessment and calculation of fungal growth inhibition. The elongation rate was calculated by averaging the changes during sequential time periods of the fungal growth. All experiments were conducted three times; a minimum of 10 conidia were examined under each treatment. Microscopic imaging was performed using a NIKON eclipse T*i* microscope (Nikon, Japan) equipped with a ProScan motorized XY stage (Prior Scientific, MA, United States) with a temperature-controlled incubator (LAUDA ECO RE 415, Korea). Bright field illumination was provided by a cool LED pE-100A (Cool LED, United Kingdom). The system is also equipped with an HF110A system, enabling rapid switching of emission wavelengths. Imaging was performed using a long working distance 10× objective (NA 0.6). Images were captured at 30 min intervals using an ANDOR zyla 5.5 MP ScMOS camera (China) and processed using the NIS elements AR 4.6 (64 bit) software package.

### Sterol Analysis

Sterol profiles of *A. flavus* (NRRL 3518) were analyzed as described previously with some modifications (Sionov et al., 2009). The samples of the fungal strain, which were grown in PDB (10^7^ conidia/ml) for 24 h at 28°C, included: (1) no drug control; (2) supplemented with 1250 μg/ml PPE; and (3) supplemented with 0.5 μg/ml PRZ. Another control sample included only PPE (1250 μg/ml) with no fungus (due to the adsorption of PPE into the mycelium that was detected following a change in the mycelium color). Three independent experiments were performed; each experiment included three biological replicates (*n* = 3) of each treatment as well as the untreated controls, with each biological replicate being one independent extraction. Mycelia were harvested by centrifugation, washed once with sterile distilled water, frozen in liquid nitrogen and lyophilized. Twenty mg of lyophilized mycelium of each sample were resuspended in 9 ml methanol; 4.5 ml 60% (wt/vol) KOH was added together with 2.5 μg cholesterol (used as an internal recovery standard). Mycelial suspension was heated to 85°C in a water bath for 2 h to complete the saponification, and the resulting mixture was cooled to room temperature. The sterols were then extracted twice with 2 ml hexane by vigorous vortex for 2 min. The upper hexane layers containing the sterols were removed, washed twice with water, and evaporated under a stream of gaseous nitrogen. Before derivatization, water residues in the sample were completely evaporated by lyophilization. Subsequently, 50 μl of N-methyl-N-(trimethylsilyl) trifluoroacetamide (MSTFA) was added, the sample was shaken vigorously, and the mixture was transferred to a 2 ml auto sampler glass vial with a 100 μl conical glass insert and analyzed by GC-MS. The GC-MS system comprised an Agilent 7890A gas chromatograph equipped with split/splitless injector, and LECO Pegasus HT Time-of-Flight Mass Spectrometer (TOFMS). GC was performed on a 30 m × 0.25 mm × 0.25 μm Rxi-5Sil MS column (Restek). Samples were analyzed in both split and splitless modes; injector and transfer line temperatures were set at 280°C. Analytes by 1 μl injected were separated using the following chromatographic conditions: Helium was used as carrier gas at a flow rate of 1.0 ml/min. The thermal gradient started at 170°C, was held at this temperature for 2 min, ramped to 280°C at 37°C/min and then ramped to 300°C at 1.5°C/min and held at 300°C for 5.0 min. Eluents were fragmented in the electron impact mode with an ionization voltage of 70 eV. The MS mass range was 50–750 m/z with an acquisition rate of 20 spectra per second. The ion source chamber was set to 230°C and the detector voltage was 1650 V. The reconstructed ion chromatograms and mass spectra were evaluated using Xcalibur software version 2.2 (Thermo Fisher Scientific). Cholesterol internal standard was identified by comparing the retention time and mass spectrometry spectra of trimethylsilyated (TMS) authentic cholesterol standard analyzed on the same instrument. The metabolites of interest were identifies by comparison with the NIST 05 Mass spectral library using NIST MS Search program v 2.3. Specifically, ergosterol (Purkait et al., 2012; Keller et al., 2015) lanosterol (Paik et al., 2008) and sitosterol (Wretensjö and Karlberg, 2002) were putatively identified based on their trimethylsilyated spectra compared with previously published spectra and relative retention times. The fragment ions at m/z 458 (M +), m/z 468 (M +), m/z 498 (M +), and m/z 486 (M +) are indicative of cholesterol, ergosterol, lanosterol and beta-sitosterol, respectively. The relative amount of each sterol was obtained by comparing the area under the curve for each sterol with that for the cholesterol internal standard in the chromatogram.

### Mycotoxin and qRT-PCR Analyses

For the evaluation of AFB1 production 100 μl of *A. flavus* inoculum (10^6^ conidia/ml) was inoculated in 25 ml PDB with PPE or PRZ alone, and in combination, and incubated at 28°C with shaking at 200 rpm up to 72 h. The samples included: (1) no drug control; (2) supplemented with 625 and 1250 μg/ml PPE; (3) supplemented with 0.0156 and 0.0312 μg/ml PRZ; and (4) supplemented with both PPE and PRZ. Another control sample included DMSO (because the tested compounds were dissolved in this solvent). After 48 and 72 h of incubation the mycelial biomass was collected by centrifugation, freeze-dried, weighed and stored at −80°C for RNA isolation. For the mycotoxin extraction procedure, the supernatant collected at the same time points was mixed with an equal volume of chloroform and vortexed for 15 min. The lower chloroform phase was dried at 50°C under a stream of gaseous nitrogen. The samples were redissolved in 300 μl of methanol and derivatized with 300 μl of trifluoroacetic acid solution (70% water, 20% trifluoroacetic acid and 10% acetic acid) for 20 min at 65°C. After 20 min 580 μl of water was added to the reacted samples. The samples were vortexed, filtered using 0.2 μm PTFE membrane filter, and quantitatively analyzed by injection of 20 μl into reverse phase UHPLC system (Agilent technologies) with a gradient elution of 0.1% acetic acid in water (59%), methanol (27%), and acetonitrile (14%) at 0.4 ml/min through a Kinetex 2.6 μm XB-C_18_ (100 × 2.1 mm) with a security guard column C18 (4 × 2 mm; Phenomenex, United States). AFB1 peaks were detected with fluorescence detector (excitation at 365 nm and emission at 455 nm) and quantified by comparing with calibration curves of the standard mycotoxin.

The total RNA was extracted from 100 mg of lyophilized mycelia of the selected samples using the Hybrid-R RNA isolation kit (GeneAll, Seoul, South Korea) according to the manufacturer’s protocol. The DNase and reverse-transcription reactions were performed on 300 ng of total RNA with the Maxima First-Strand cDNA Synthesis Kit (Thermo Fisher Scientific, Waltham, MA, United States) according to the manufacturer’s instructions. The cDNA samples were diluted 1:5 (v/v) with ultrapure water. The quantitative real time PCR was performed using Fast SYBR green Master Mix (Applied Biosystems, Waltham, MA, United States) in a StepOnePlus Real-Time PCR System (Applied Biosystems, Waltham, MA, United States). The primer pairs for the specific 3 genes involved in aflatoxin biosynthesis (*aflR*, *aflC*, and *aflD*) were synthesized based on previous studies (Zhao et al., 2018; Lan et al., 2019). The PCR conditions were as follows: 95°C for 20 s, followed by 40 cycles of 95°C for 3 s and 60°C for 20 s. The sample was normalized using β-actin and the relative expression levels were measured using the 2^(–Δ^ ^Δ^ ^Ct)^ analysis method. Results were analyzed with StepOne software v2.3.

### Statistical Analysis

The data were presented as the means ± SE (standard error) of three experiments by measuring three independent replicates. An unpaired *t*-test was used to compare differences in gene expression level between *A. flavus* isolate treated with the compounds and the untreated (no drug) control. A *p-*value of less than 0.05 was considered to be significant (^∗^*p* < 0.05, ^∗∗^*p* < 0.01).

## Results and Discussion

### Synergistic Antifungal Activity of PPE With Azole Drugs

*In vitro* susceptibility of several important mycotoxigenic species, including *A. flavus*, *A. parasiticus*, *A. fumigatus*, *F. proliferatum* and *F. verticillioides*, to a PPE extract and azole antifungal agent was examined. The pomegranate extract was found to be active against the fungal isolates at relatively high concentrations, with MIC values between 1.25 and 5 mg/ml (Table 1). These values are in good agreement with those previously reported for PPE on its *in vitro* and *in vivo* antifungal effect against major fungal post-harvest pathogens (Li Destri Nicosia et al., 2016; Pangallo et al., 2017). Agricultural antifungal azole drug, PRZ, had considerably lower MIC values in the range of 0.25 to 1 μg/ml. Following determination of the individual MIC values, the efficacy of PPE in combination with the azole compound was examined by checkerboard assays against the fungal isolates. Despite its apparent low antifungal activity when used alone, PPE demonstrated synergistic inhibitory effects when combined with PRZ against *A. flavus*, *A. fumigatus* and *F. proliferatum*, with FICI values of 0.25 to 0.5 (Table 1); additive effects were observed against *A. parasiticus* and *F. verticillioides* (FICI 0.75–1). It is noteworthy that in the presence of PPE the MIC values of PRZ for *A. flavus* and *F. proliferatum* were reduced by 8- and 16-fold, respectively. Moreover, PRZ lowered the MIC values of PPE by four- to eight-fold against same isolates. These results indicate that the combined approach may decrease the required concentrations of the compounds to effectively inhibit mycotoxigenic fungi. Such combination may allow lower doses of the agents to be used in any application while reducing potential concerns over dosage levels and/or non-specific toxicity of the single compound.

**TABLE 1:** *In vitro* susceptibility of mycotoxigenic fungi to PPE and PRZ and in combination.

**Fungal strains**	**MICs of compounds (μg/ml)**				**FICIs**	**Interpretation**
			
	**Alone**		**In combination**			
				
	**PPE**	**PRZ**	**PPE**	**PRZ**		
*A. flavus* (NRRL 3518)	2500	0.25–0.5	625	0.0625	0.37	Synergy
*A. parasiticus* (NRRL 6111)	2500	0.5–1	1250	0.25	0.75	Additive
*A. fumigatus* (NRRL 62427)	2500	0.25–0.5	312.5	0.0625	0.5	Synergy
*F. verticillioides* (NRRL 25457)	5000	0.25–0.5	2500	0.125	1	Additive
*F. proliferatum* (NRRL 31866)	5000	0.5–1	625	0.0625	0.25	Synergy

*PPE, pomegranate peel extract; PRZ, prochloraz; MIC, minimum inhibitory concentration; FICI, fractional inhibitory concentration index.*

### Live-Imaging Based Investigation of the Inhibitory Effects of PPE/PRZ on Mycotoxigenic Fungi

**FIGURE 1:**
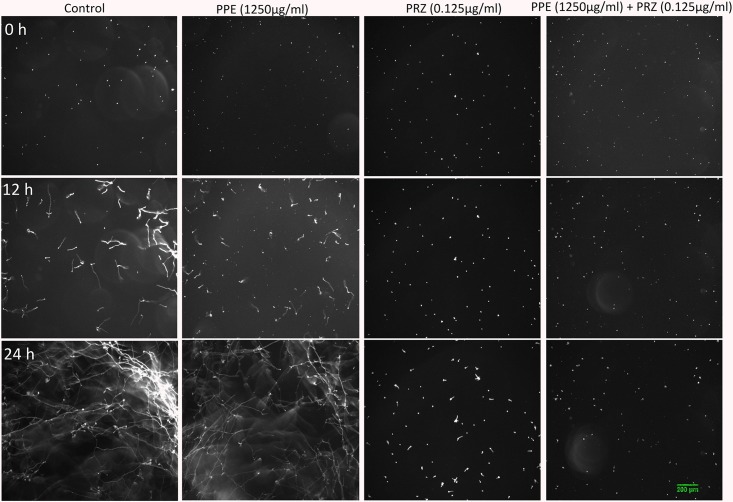
Time-lapse microscopic images of *A. flavus* treated with antifungal compounds. Three different time points as indicated are given to visualize the images of *A. flavus* treated with PPE/PRZ alone and in combination (original magnification: ×10). Experiments were repeated three times and results of a single representative experiment are shown.

**FIGURE 2:**
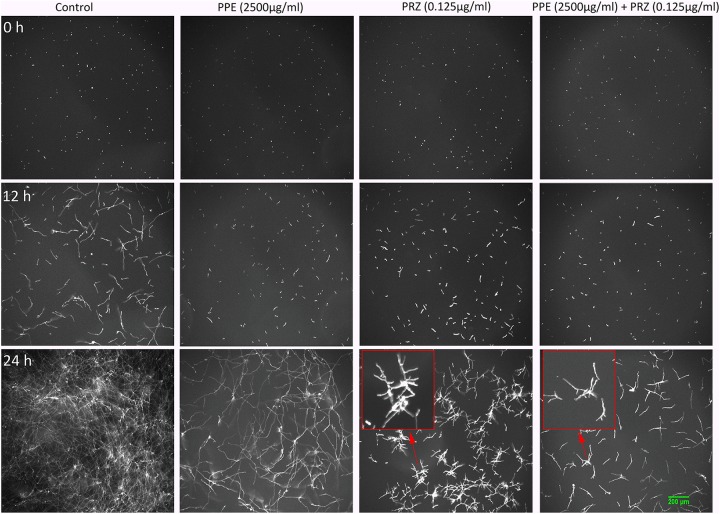
Time-lapse microscopic images of *F. proliferatum* treated with antifungal compounds. Three different time points as indicated are given to visualize the images of *F. proliferatum* treated with PPE/PRZ alone and in combination (original magnification: ×10). Enlarged regions of the original images are marked with red box. Experiments were repeated three times and results of a single representative experiment are shown.

Using time-lapse microscopy we could clearly demonstrate the effect of each compound alone, as well as their combination, on the kinetics of conidial germination, hyphal growth, and branch initiation in *A. flavus* and *F. proliferatum*. One of the most striking features of the time-lapse sequences obtained from our experiments is the apparent delay in germination following treatment with PPE at two-fold lower concentration than its MIC, with both fungi showing markedly reduced growth at 12 h compared to untreated control (Figures 1, 2 and Supplementary Videos S1, S2). However, after 24 h of incubation the growth of PPE-treated *A. flavus* (1250 μg/ml) was not clearly distinguishable from the untreated control. Treatment of *A. flavus* with the azole drug at sub-MIC concentration (0.125 μg/ml PRZ) proved highly effective, resulting in marked inhibition of both germination and growth over 24 h of incubation. Interestingly, a combined treatment (1250 μg/ml PPE + 0.125 μg/ml PRZ) resulted in further reduction in both parameters (Figure 1 and Supplementary Video S1). A different image emerged for *F. proliferatum*, where PPE effect at 2500 μg/ml resulted in significant delay of branch initiation and reduction of lateral branches formation compared to untreated control over the course of the experiment (Figure 2 and Supplementary Video S2). Surprisingly, over the 1st 12 h of incubation, PPE treatment appeared to be more effective than treatment with 0.125 μg/ml PRZ. Following 24 h of incubation, however, PRZ treatment resulted in stunted growth of *F. proliferatum* characterized by severe hyper-branching. Similar to *A. flavus*, the combined treatment here (2500 μg/ml PPE + 0.125 μg/ml PRZ) appeared to be the most effective, resulting in the least observed growth while also eliminating the hyper-branching observed for PRZ alone, suggesting the two treatments to have different modes of action and possibly synergistic effects. Interestingly, while reduced growth rate is many times accompanied by hyper-branching, as in the case of PRZ effect on *F. proliferatum*, hyphal polar extension and hyphal branching are two distinct morphological processes which are independently regulated (Seiler and Plamann, 2003; Ziv et al., 2009). This is further supported by the different changes induced in the growth and structure of hyphae, which indicate a different effect for both PPE and PRZ.

### Quantification of Antifungal Effects of Sub-MIC Concentrations of PPE and PRZ by Image Analysis

**FIGURE 3:**
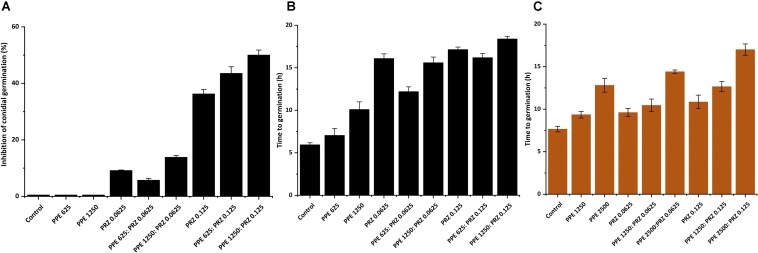
Effect of antifungal compounds on conidial germination. **(A)** Percentages of inhibition of *A. flavus* conidial germination following treatment with different concentrations of PPE and PRZ both alone and in combination. **(B)** Effect of PPE/PRZ both alone or in combination on time required for germination of *A. flavus* conidia, and **(C)** *F. proliferatum* conidia. Results are expressed as the means ± SE of three experiments.

**FIGURE 4:**
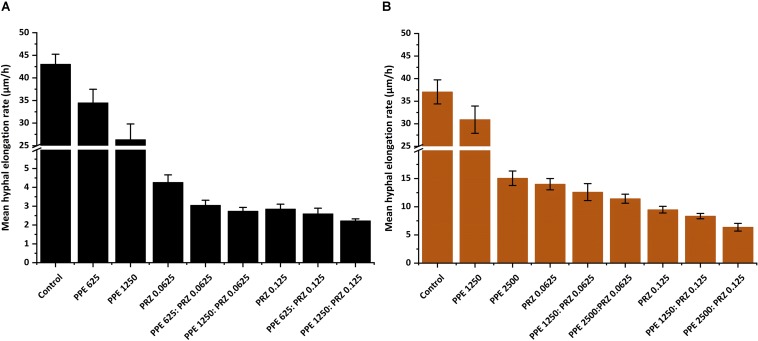
Graphical representation of the fungal growth under treatment with antifungal compounds. Mean hyphal elongation rates of **(A)** *A. flavus*, and **(B)** *F. proliferatum* treated with different concentrations of PPE and PRZ both alone and in combination. Results are expressed as the means ± SE of three experiments.

A major advantage of live-imaging microscopy over more traditional approaches for measuring fungal growth is the ability to observe and quantify the growth parameters of individual spores. The usefulness of this approach has been demonstrated in a number of studies, and was applied for monitoring different dynamic processes in bacteria and fungi including sporulation, mycelial growth, and host-pathogen interactions (Löfman et al., 1997; Richter et al., 2007; Jyothikumar et al., 2008; Held et al., 2010, 2011; Grünberger et al., 2017; Marshall et al., 2017). Here we utilize the power of this approach to quantitatively compare the effect of conventional and natural antifungal agents on several growth parameters of *A. flavus* and *F. proliferatum*. Conidia or sexual spores are critical in the fungal life cycle. Fungal development starts from conidial germination under favorable growth conditions. Therefore, the dynamic analysis of early germination under treatments with antifungal compounds might improve our understanding of the physiological response of fungi and thus may help for the development a new combination strategy to fight fungal infections. An antifungal treatment may reduce fungal biomass by delaying spore germination or by reducing the overall rate of germination (i.e., percent of spores that develop into hyphae). Treatment with PPE alone did not affect germination rate in *A. flavus* regardless of concentration. The effect of PRZ treatment was dose dependent, with approximately 9% reduction in germination rate for 0.0625 μg/ml compared to 36% inhibition for 0.125 μg/ml (Figure 3A and Supplementary Figure S1A). Moreover, the combinations of PPE with PRZ at 0.125 μg/ml resulted in higher rates of inhibition, with germination rate dropping to 50%. Different from *A. flavus, F. proliferatum* conidia showed 100% germination for all treatments, pointing out that any treatment only delayed conidial germination but did not alter the overall germination rate (Supplementary Figure S1B). The mean time to germination, determined as the time point at which the germ tube exceeded spore diameter, was found to be affected by all tested treatments. Mean time to germination for the untreated *A. flavus* control was 5.95 h, compared to 10.1 h following treatment with 1250 μg/ml PPE and 17.15 h following treatment with 0.125 μg/ml PRZ (Figure 3B). Similarly, the untreated *F. proliferatum* control had a mean time to germination of 7.65 h, while treatment with 2500 μg/ml PPE extended the mean germination time to 12.8 h (Figure 3C). Interestingly, treatment with 0.125 μg/ml PRZ resulted in a less pronounced delay, with a mean time to germination of just 10.85 h. The effect of combined treatments on the mean time to germination in *A. flavus* varied with concentration. No synergy between the two treatments was found when 625 μg/ml PPE where added to either concentration of PRZ (Figure 3B). Addition of 1250 μg/ml PPE had no effect on germination time when combined with 0.0625 μg/ml PRZ, compared to the azole drug alone, but had a slight positive effect when combined with 0.125 μg/ml PRZ, extending the mean time to germination from 17.15 h for PRZ alone to 18.4 h for the combined treatment. A better synergistic inhibitory effects were found for combined treatments against *F. proliferatum*, especially when either PRZ concentration was combined with 2500 μg/ml PPE, extending the time to germination up to 17 h (Figure 3C). Hyphal elongation rate is another criterion used to determine the effect of the compounds on fungal growth while using live imaging microscopy techniques. PPE treatment at a concentration of 1250 μg/ml slowed the mean elongation rates of both *A. flavus* and *F. proliferatum* hyphae up to 26.34 and 30.9 μm/h, respectively, compared to those of the untreated controls (43.04 and 37.06 μm/h, respectively) (Figures 4A,B). When *A. flavus* was treated with the azole drug at sub-MIC concentrations of either 0.0625 or 0.125 μg/ml, the mean elongation rate of the filaments was nearly 10-fold slower (2.85–4.26 μm/h) compared to that of the untreated control. It is noteworthy that the mean hyphal elongation rate for *A. flavus* almost unchanged under combination treatment compared to the azole drug alone (Figure 4A). The pomegranate extract at two-fold higher concentration (2500 μg/ml) significantly inhibited *F. proliferatum* growth and led to reduction in the mean of hyphal elongation rate up to 15.05 μm/h. Under PRZ treatment *F. proliferatum* hyphal mean elongation rate considerably reduced up to 75% (9.47 μm/h) in comparison to the control (Figure 4B). However, the combined treatment of PPE with PRZ, when tested at higher sub-MIC concentrations, yielded synergistic inhibitory effect with further reduction of *F. proliferatum* hyphal extension rate up to 6.36 μm/h compared to each compound alone (Figure 4B). Following germination, the elongation rate of the hypha increases exponentially toward a maximum linear rate (Robson, 1999), which is an additional measurement standard that has been calculated in order to evaluate antifungal efficacy of the compounds and their combinations. Interestingly, unlike the mean elongation rate, the maximum rate of hyphal extension for both fungi was lower under combination treatment at higher sub-MIC concentrations of the compounds compared to the azole drug alone (Supplementary Figures S2A,B). These data are reflected in video-microscopy images where synergistic effect of the PPE compound combined with the azole drug PRZ was clearly observed (Figures 1, 2). As was expected, hyphal elongation rate is closely correlated with the time required for conidial germination: the longer it takes to germinate due to the treatment, the lower the rate of the hyphal extension.

### Effect of PPE on Sterol Composition in *A. flavus*

**FIGURE 5:**
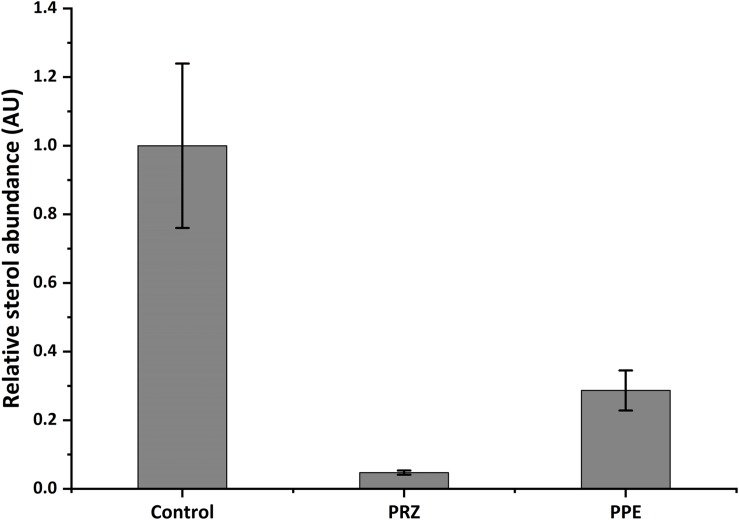
Relative abundance of ergosterol in *A. flavus* as detected by GC-MS. Data presented is peak area of ergosterol relative to cholesterol standard, normalized to control. Results are expressed as the average ± SE of three independent experiments, each with three biological replicates; AU, arbitrary units.

Ergosterol is a lipid responsible for fungal cell membrane fluidity and permeability and plays a crucial role in its viability. Several antifungals, such as azole drugs, primarily target ergosterol biosynthesis. Azole antifungals affect ergosterol biosynthesis via inhibition of 14-α demethylase (Cyp51/Erg11), a fungal cytochrome P-450 enzyme, which mediates the conversion of lanosterol to ergosterol. Application of PRZ treatments to *Sclerotinia sclerotiorum* and *Botrytis cinerea* have indicated that sterol-inhibiting fungicides do not inhibit spore germination or initial cell growth but result in aberrant hyphal morphology, namely swollen hyphae and/or hyper-branching (Pappas and Fisher, 1979; Zhang et al., 2018) similar to our observation in *F. proliferatum* in the current work. This is also in line with the phenotypic consequence of mutating *erg11* (or *cyp51*, the suspected target of PRZ). A conditional mutant of *Neurospora crassa* demonstrate that Erg11 is required for hyphal elongation but not germination (Hu et al., 2018). However, the pronounced effect of PRZ on *A. flavus* germination and the synergistic effect with PPE may suggest a more complex effect of these drugs on the fungal physiology. We hypothesized that PPE, similarly to PRZ, may interfere with ergosterol function in the fungal cell membrane. To test this hypothesis, the sterol profile of *A. flavus* was compared with that of *A. flavus* treated with PPE and/or azole antifungal PRZ. Without any drug treatment, ergosterol was the major fraction of the total sterol content (Figure 5 and Supplementary Figure S3). Following PRZ treatment, the ergosterol content was dramatically reduced in *A. flavus* in parallel to an increase of lanosterol (Supplementary Figure S3), consistent with the classic pattern of *Aspergillus* sterol 14α-demethylase activity following treatment with azoles (Parker et al., 2014). Interestingly, PPE treatment of the isolate also resulted in a significant decrease of ergosterol content in comparison to the untreated control (Figure 5). No lanosterol was detected in the PPE treatment, however, this could be due to the wide peak of β-sitosterol which co-eluted with lanosterol and was highly abundant in the PPE itself, as indicated by the PPE control samples (blank, without fungus) (Supplementary Figure S3). The results suggest that PPE compound may act upstream or downstream of the azole target Erg11 and might interfere with sterol function through potential inhibition of certain enzymatic steps in the ergosterol biosynthesis pathway.

### Anti-mycotoxigenic Activity of PPE/PRZ

**FIGURE 6:**
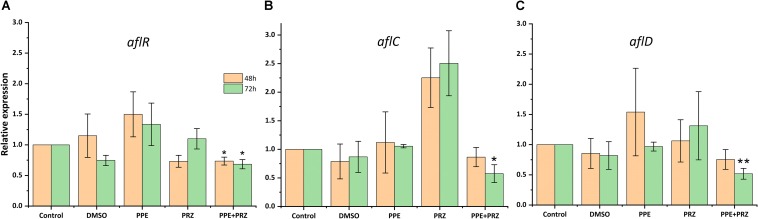
Effect of antifungal compounds on the expression of key aflatoxin biosynthetic pathway genes in *A. flavus*. Expression ratios of **(A)** *aflR* (aflatoxin transcription factor), **(B)** *aflC* (polyketide synthase), and **(C)** *aflD* (nor-1/reductase) in *A. flavus* treated with PPE (625 μg/ml) and PRZ (0.0156 μg/ml) both alone and in combination compared to untreated (no drug) control; DMSO control was also included. The expression of each gene in the untreated (no drug) control samples was normalized as 1.0. Results are expressed as the average ± SE of three independent experiments, each with three biological replicates, ^∗^*p* < 0.05, ^∗∗^*p* < 0.01. SE in normalized control (no drug) samples is 0 due to the 2^(–Δ^ ^Δ^ ^Ct)^ analysis method.

Pomegranate peel extract treatments appear to have the capability of inhibiting aflatoxin production by *A. flavus* (Table 2). In particular, after 72 h of incubation the compound at the concentration of 1250 μg/ml inhibited AFB1 production by 67% without affecting the fungal growth. Reduction in fungal biomass by at least 50% was observed under PPE treatment at the higher concentration of 2500 μg/ml with subsequent inhibition of AFB1 production by 97%. These findings suggest that PPE inhibitory activity of fungal growth and mycotoxin formation are not directly related, and the inhibition of aflatoxin production by the extract could involve an inhibition of specific enzymes in the pathway of aflatoxin biosynthesis. Compared to the untreated controls, there was an increase in AFB1 production by the fungus when treated with an azole agent PRZ or pomegranate extract at low concentrations of 0.0156 and 625 μg/ml, respectively (Table 2). Among different environmental factors, such as temperature, oxidative stress, water activity and pH, application of low fungicide concentrations might be an additional stress factor stimulating mycotoxin biosynthesis by fungi as a defense response. Several studies reported that sub-lethal concentrations of synthetic or natural fungicides stimulated mycotoxin production. For example, increased production of deoxynivalenol (DON) and 3-acetyl deoxynivalenol (3-ADON) has been observed when low doses of azole fungicides were used against *F. culmorum* cultures (D’Mello et al., 1998; Magan et al., 2002). A four-fold increase in aflatoxin synthesis by *A. parasiticus* on different substrates occurred in the presence of sub-inhibitory level of miconazole (Buchanan et al., 1987). A number of studies reported that several plant essential oils at sub-lethal concentrations could reduce the growth of mycotoxigenic *Fusarium* and *Aspergillus* species, but stimulated their toxins production (Hope et al., 2005; Nerilo et al., 2016; Morcia et al., 2017). However, in the current study, combination of two compounds at suboptimal concentrations completely inhibited AFB1 synthesis by *A. flavus*, compared to increased mycotoxin production by the fungus when treated with each compound alone (Table 2). Furthermore, the effect of PPE, PRZ and their combination at suboptimal concentrations on the expression level of key genes in the aflatoxin biosynthesis cluster, *aflR* (aflatoxin transcription factor), *aflC* (polyketide synthase) and *aflD* (nor-1/reductase), was analyzed by qRT-PCR. The results indicated that the expression levels of these genes were down-regulated under combined treatment of PPE with PRZ at low doses, directly causing depression of aflatoxin production (Figure 6). These findings are consistent with a recent study of Wang et al. (2018) where combination of cinnamaldehyde and citral (the major components of *Cinnamon bark* essential oil) at sub-MIC concentrations resulted in a significant decrease of patulin biosynthesis by *Penicillium expansum*. According to the RNA sequencing results in that study, the expressions of all the 15 genes involved in patulin biosynthetic pathway were down-regulated under cinnamaldehyde and citral combined treatment at sub-MIC concentrations (Wang et al., 2018). Taking these results together, we suggest that the complete elimination of the mycotoxin can be achieved by an azole fungicide application at very low concentrations with less toxicity to the environment when combined with PPE, through modulating the expression of key aflatoxin biosynthetic pathway genes.

**TABLE 2:** Effect of antifungal compounds and their combination on aflatoxin B1 production by *A. flavus.*

**Compounds^a^**	**AFB1 (ng/ml)**	
	
	**48 h incubation**	**72 h incubation**
Control 1 (no treatment)	65.33 ± 2.1^b^	147.79 ± 6.18
Control 2 (DMSO)	73.87 ± 3.42	157.37 ± 5.94
PPE 625 μg/ml	nd	188.84 ± 6.63
PPE 1250 μg/ml	nd	48.97 ± 4.22
PRZ 0.0156 μg/ml	nd	217.57 ± 11.7
PRZ 0.0312 μg/ml	nd	nd
PPE 625 + PRZ 0.0156	nd	nd
PPE 625 + PRZ 0.0312	nd	nd

*^*a*^No reduction in fungal biomass was observed under treatment with each compound alone and in combination at the concentrations indicated in the table; ^*b*^Average values of mycotoxin concentration ± (SE) standard error (the average of three independent experiments, each with three biological replicates) “nd” not detected.*

## Conclusion

In summary, our study demonstrated that the combination of natural antifungal compound with conventional synthetic fungicide is highly effective at inhibiting growth of certain major mycotoxigenic and food-spoilage fungi. This combination was particularly effective while producing a synergistic suppression effect at considerably lower doses on aflatoxin biosynthesis by *A. flavus*. Moreover, the results may provide flexibility to determine the dose range of the compounds that can be used in combination for practical applications. Therefore, it is proposed that this combination approach can offer an effective strategy for controlling fungal growth and mycotoxin production in agricultural commodities.

## Data Availability

The raw data supporting the conclusions of this manuscript will be made available by the authors, without undue reservation, to any qualified researcher.

## Author Contributions

SS, OS, and ES conceived and designed the experiments. SS, CZ, OB, and VZ performed the experiments. SS, OS, CZ, OB, and ES analyzed the data. SS, OS, CZ, and ES wrote the manuscript. All authors read and approved the final manuscript.

## Conflict of Interest Statement

The authors declare that the research was conducted in the absence of any commercial or financial relationships that could be construed as a potential conflict of interest.
