# Supplementary material for: Synergistic Inhibition of Mycotoxigenic Fungi and Mycotoxin Production by Combination of Pomegranate Peel Extract and Azole Fungicide
Source: Front Microbiol. 2019 Aug 20;10:1919. doi: 10.3389/fmicb.2019.01919 (PMC6710344; doi:10.3389/fmicb.2019.01919)
Supplement: Supplementary file 1 [file Data_Sheet_1.docx]

Supplementary Material

**Supplementary figures**

**A**

**B
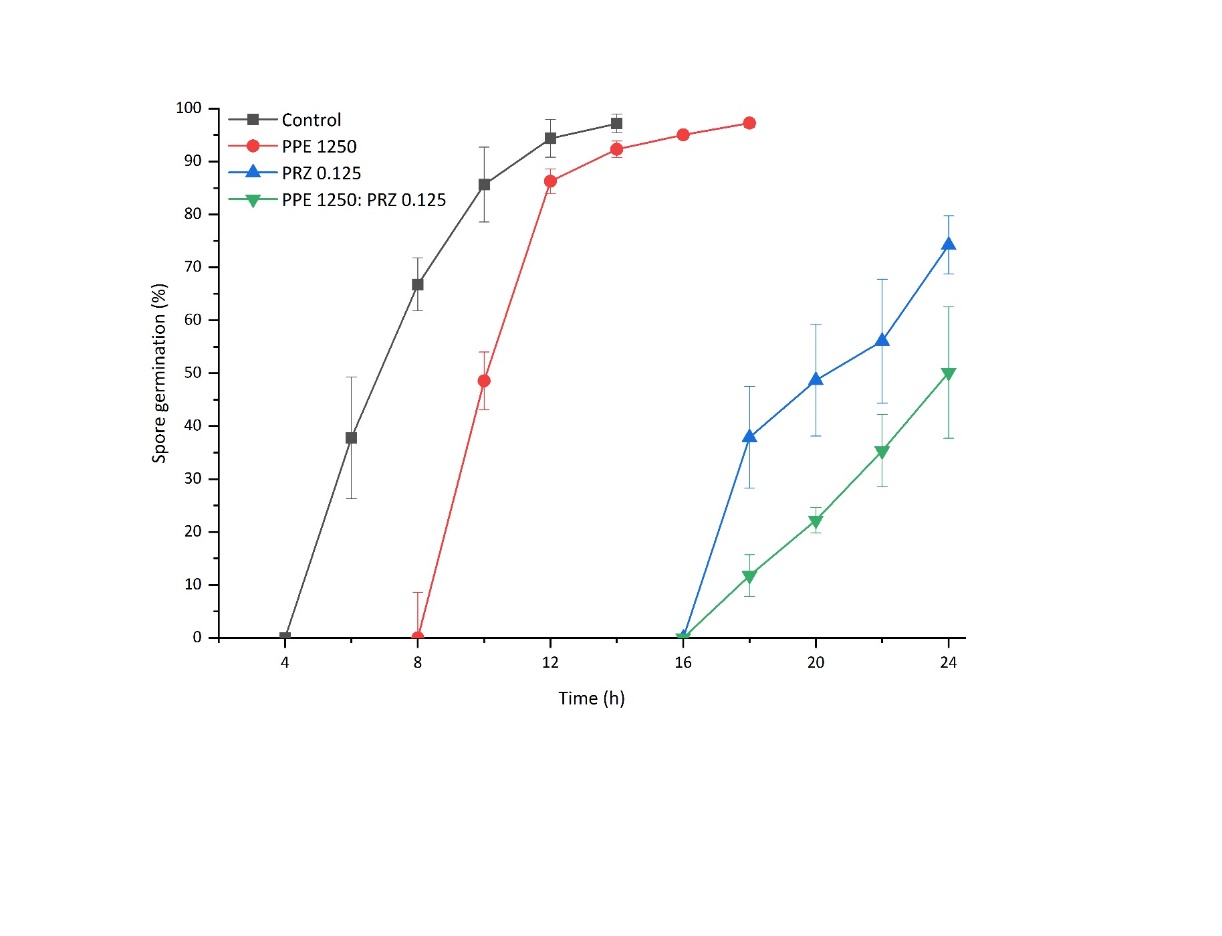
**

**
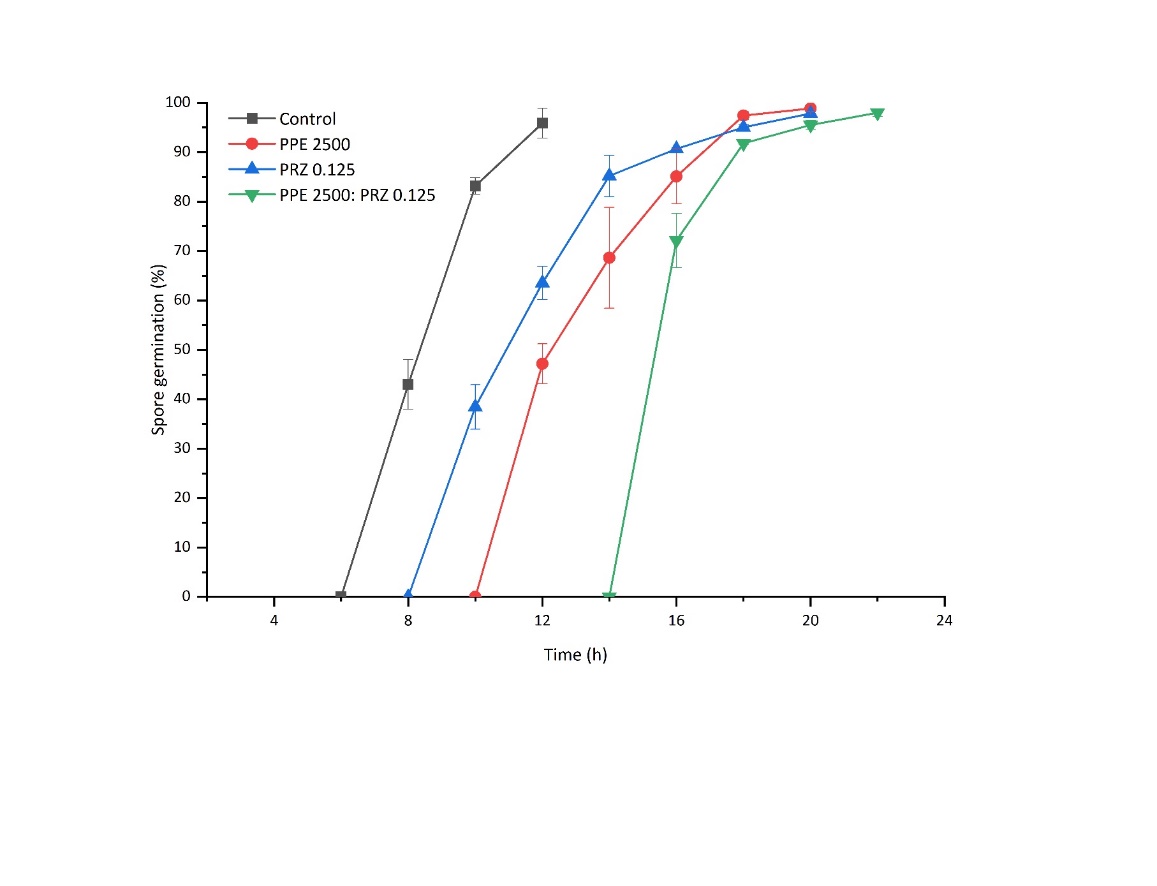
**

**FIGURE S1. Kinetics of fungal conidia germination*.*** Percentages of germination of conidia over time in **(A)** *A. flavus,* and **(B)** *F. proliferatum* following treatment with PPE and PRZ both alone and in combination. Experiments were repeated three times and results are expressed as the means ± SD of three experiments.

**A**

**
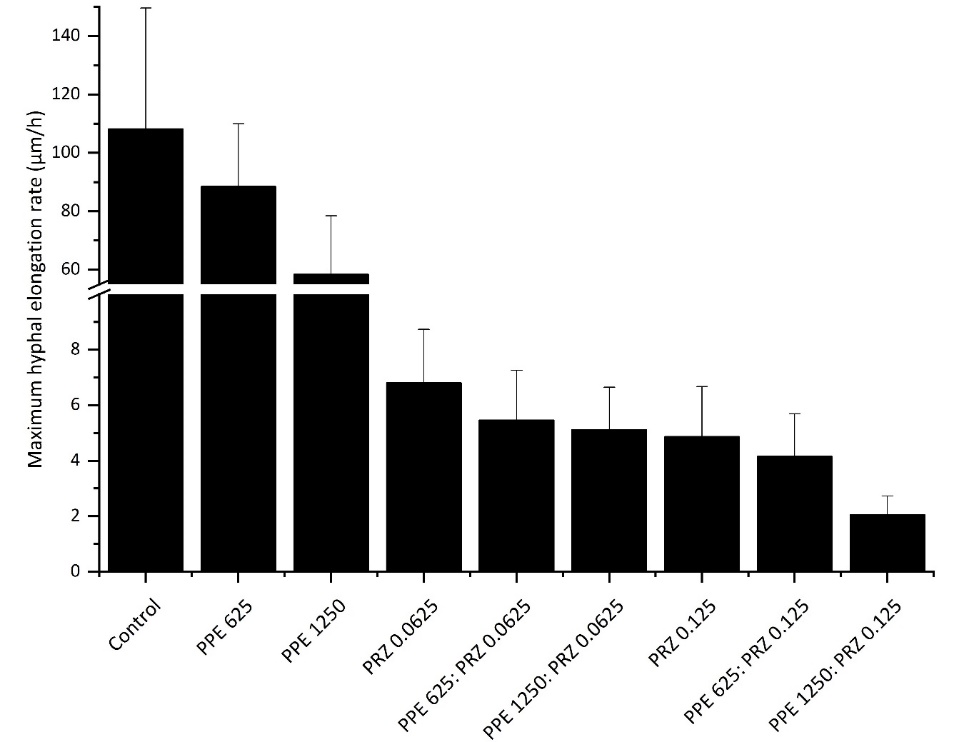
**

**B**

**
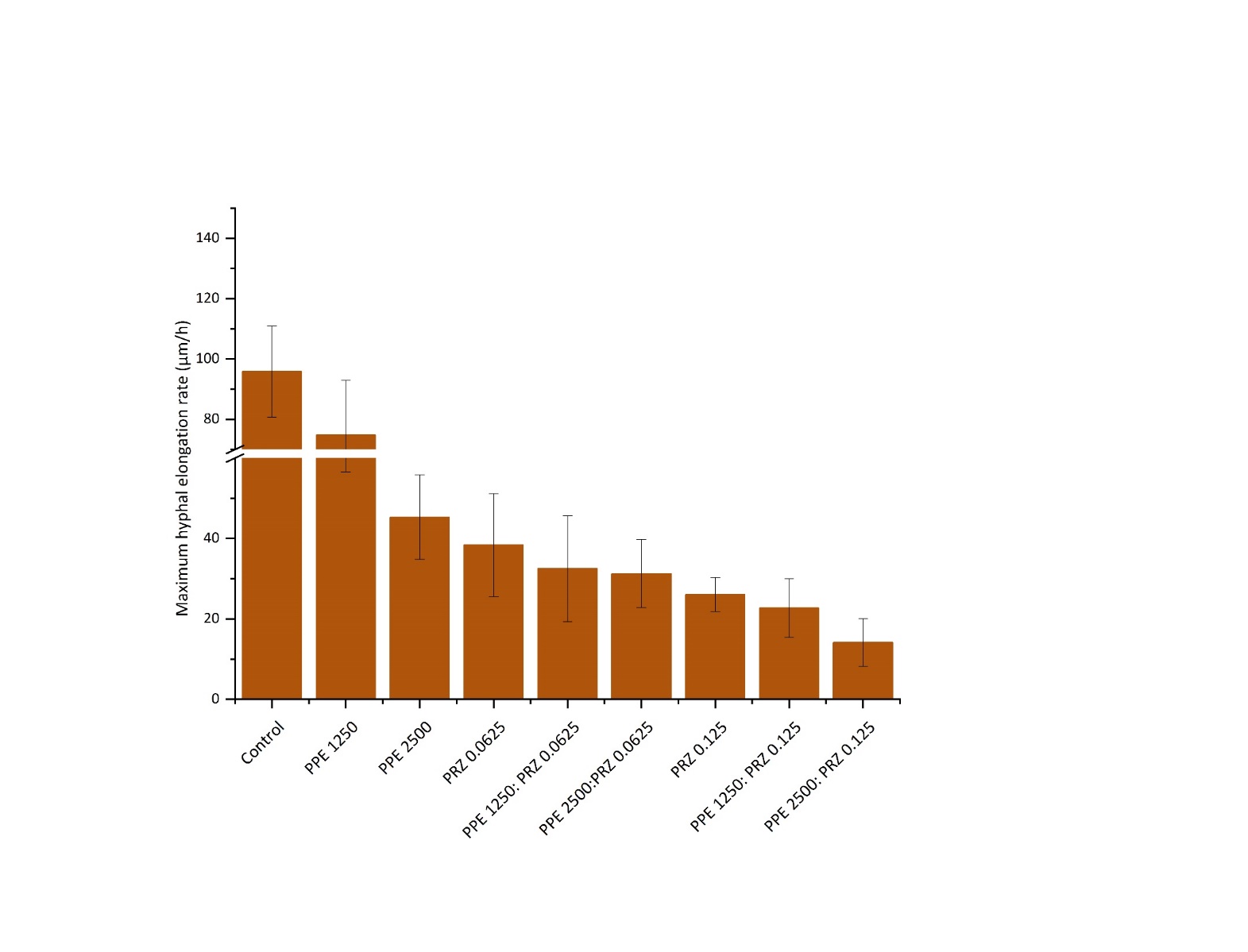
**

**FIGURE S2. Maximum rates of hyphal extension** for **(A)** *A. flavus,* and **(B)** *F. proliferatum* treated with different concentrations of PPE and PRZ both alone and in combination. Results are expressed as the means ± SD of three experiments.

**A B**

**
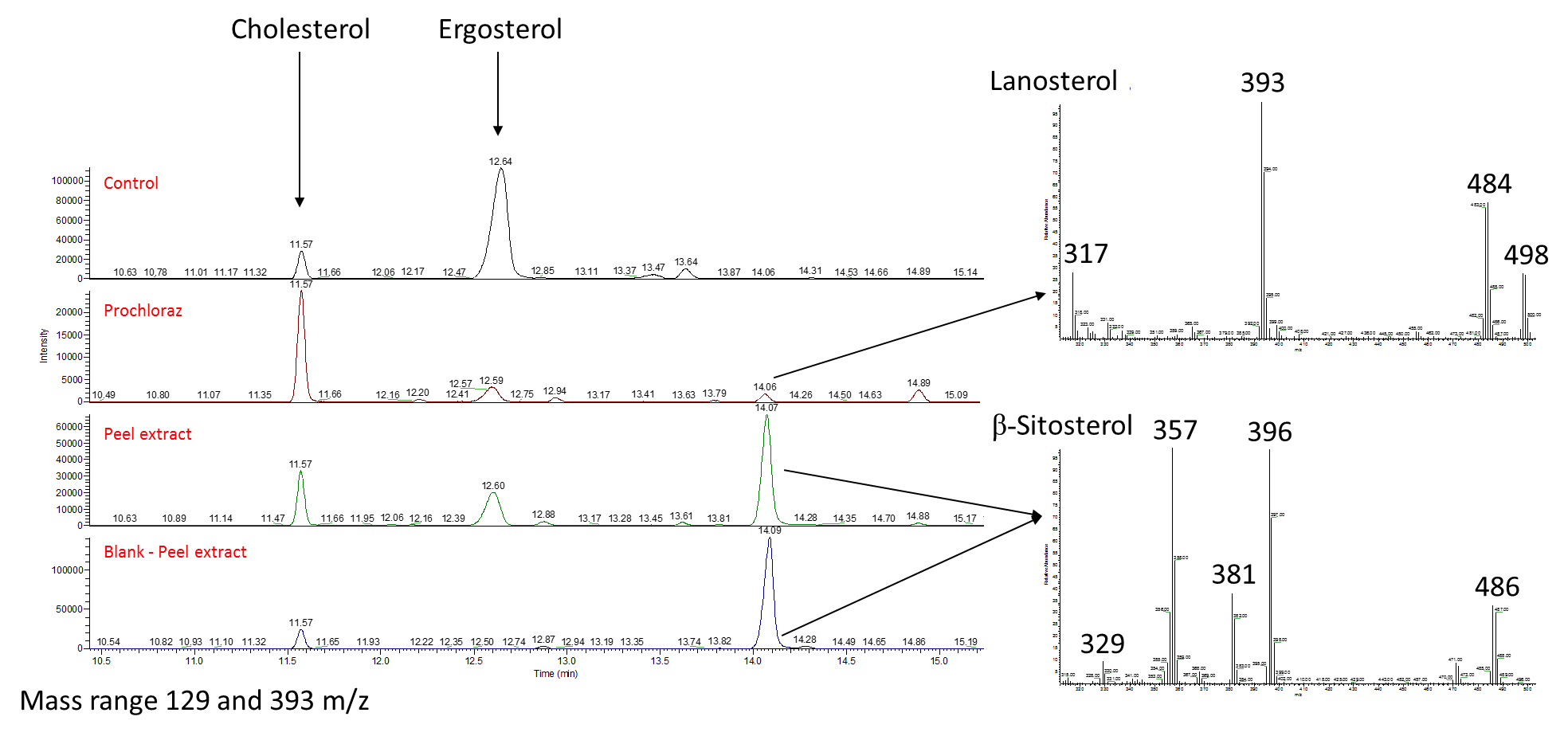
**

Intensity

**C**

**
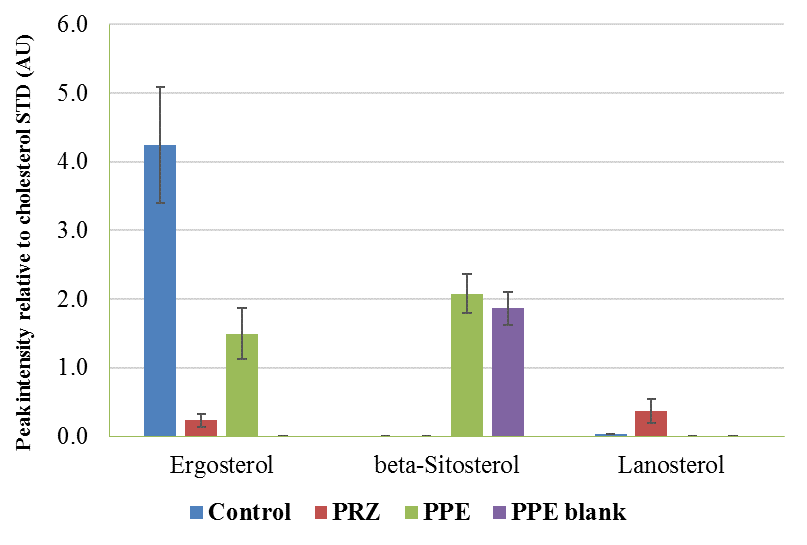
**

**FIGURE S3. GC-MS analysis of *A. flavus* sterol profile.** The samples of *A. flavus* were grown without any drug (control), with the presence of 0.5 µg/ml PRZ or 1250 µg/ml PPE. Another control sample included only PPE (1250 µg/ml) with no fungus (due to the adsorption of PPE into the mycelium that was detected following a change in the mycelium color). Total sterols were extracted from each sample and analyzed by GC-MS. Inhibition of ergosterol biosynthesis by PRZ resulted in the accumulation of several sterols intermediates (RT= 12.9, 14.06 lanosterol and 14.89). PPE induced only partial reduction in ergosterol accumulation, which did not lead to a detectable sterols intermediates. Specifically, lanosterol could not be detected in PPE treatment, possibly due to its co-elution with sitosterol (at RT 14.09 min) (Mackay, et al. 2014; Li et al., 2007) which is highly abundant in the peel extract (Wu and Tian, 2017) and probably adsorb into the fungal hyphae. Putative identification of the sterols was performed according to their full spectra, against NIST library. **(A)** Chromatograms (GC-MS) presenting fragment m/z 129 and 393, which are known fragment ions of the TMS derivatives of the identified sterols (Gwatidzo et al., 2014). Experiments were repeated three times and results of a single representative experiment are shown. **(B)** Positive ion mass spectra at retention time 14.09 min is indicative of TMS derivatives of lanosterol in PRZ treatment (top spectrum) vs. β-sitostreol in PPE treatment and PPE blank samples (bottom spectrum) **(C)** Relative abundance of the sterols in *A. flavus* as detected by their TMS derivatives. Results are expressed as the means ± SD of three experiments performed in triplicate.

**References**

Gwatidzo, L., Botha, B. M., McCrindle, R. I., and Combrinck, S. (2014). Extraction and identification of phytosterols in manketti (*Schinziophyton rautanenii*) nut oil. *JAOCS, J. Am. Oil Chem. Soc.* 91, 783–794. doi:10.1007/s11746-014-2417-2.

Li, T. S. C., Beveridge, T. H. J., and Drover, J. C. G. (2007). Phytosterol content of sea buckthorn (*Hippophae rhamnoides* L.) seed oil: Extraction and identification. *Food Chem.* 101, 1633–1639. doi:10.1016/j.foodchem.2006.04.033.

Mackay, D. S., Jones, P. J. H., Myrie, S. B., Plat, J., and Lütjohann, D. (2014). Methodological considerations for the harmonization of non-cholesterol sterol bio-analysis. *J. Chromatogr. B Anal. Technol. Biomed. Life Sci.* 957, 116–122. doi:10.1016/j.jchromb.2014.02.052.

Wu, S., and Tian, L. (2017). Diverse phytochemicals and bioactivities in the ancient fruit and modern functional food pomegranate (*punica granatum*). *Molecules* 22. doi:10.3390/molecules22101606.
